# Brewers’ spent grain as substrate for dextran biosynthesis by *Leuconostoc pseudomesenteroides* DSM20193 and *Weissella confusa* A16

**DOI:** 10.1186/s12934-021-01515-4

**Published:** 2021-01-22

**Authors:** Prabin Koirala, Ndegwa Henry Maina, Hanna Nihtilä, Kati Katina, Rossana Coda

**Affiliations:** 1 Department of Food and Nutrition, University of Helsinki, 00014 Helsinki, Finland; 2 Helsinki Institute of Sustainability Science, Helsinki, Finland

**Keywords:** Brewers’ spent grain, Lactic acid bacteria, Fermentation, Dextran

## Abstract

**Background:**

Lactic acid bacteria can synthesize dextran and oligosaccharides with different functionality, depending on the strain and fermentation conditions. As natural structure-forming agent, dextran has proven useful as food additive, improving the properties of several raw materials with poor technological quality, such as cereal by-products, fiber-and protein-rich matrices, enabling their use in food applications. In this study, we assessed dextran biosynthesis in situ during fermentation of brewers´ spent grain (BSG), the main by-product of beer brewing industry, with *Leuconostoc pseudomesenteroides* DSM20193 and *Weissella confusa* A16. The starters performance and the primary metabolites formed during 24 h of fermentation with and without 4% sucrose (w/w) were followed.

**Results:**

The starters showed similar growth and acidification kinetics, but different sugar utilization, especially in presence of sucrose. Viscosity increase in fermented BSG containing sucrose occurred first after 10 h, and it kept increasing until 24 h concomitantly with dextran formation. Dextran content after 24 h was approximately 1% on the total weight of the BSG. Oligosaccharides with different degree of polymerization were formed together with dextran from 10 to 24 h. Three dextransucrase genes were identified in *L. pseudomesenteroides* DSM20193, one of which was significantly upregulated and remained active throughout the fermentation time. One dextransucrase gene was identified in *W. confusa* A16 also showing a typical induction profile, with highest upregulation at 10 h.

**Conclusions:**

Selected lactic acid bacteria starters produced significant amount of dextran in brewers’ spent grain while forming oligosaccharides with different degree of polymerization. Putative dextransucrase genes identified in the starters showed a typical induction profile. Formation of dextran and oligosaccharides in BSG during lactic acid bacteria fermentation can be tailored to achieve specific technological properties of this raw material, contributing to its reintegration into the food chain.

## Background

Brewers’ spent grain (BSG), the solid fraction obtained from malted barley after filtration during the beer brewing process is a very abundant side stream generated by the food industry [1]. Its production is estimated to be 39 million tons globally and accounts for ca. 85% of total waste generated during the beer brewing process [2, 3]. BSG is a lignocellulosic material containing up to 50% fiber (hemicellulose and cellulose) and 30% protein (w/w) and up to 28% lignin (w/w) of dry matter [3], whose composition varies with the variety of barley and conditions used during the malting process [1]. Currently, BSG is used mainly for feed and energy purposes, however, its abundant availability at low cost and its richness in fibers and proteins make it a valuable material for food applications [3].

The main reasons for poor reutilization of BSG in food are the technological challenges deriving from its peculiar composition and structure, resulting in unwanted sensory attributes and impaired food quality [3]. Milling and bioprocessing methods along with fermentation have shown positive influence on these factors, coping with the difficult aspects of reintegrating BSG into the food chain [4, 5]. Lactic acid bacteria (LAB) fermentation is an effective approach to enhance the nutritional and techno-functional property of technologically challenging raw materials, facilitating their use in food applications. Among the well-known benefits of LAB fermentation, the synthesis of exopolysaccharides in situ successfully modified the functionality of cereal by-products, fiber-and protein-rich matrices [6, 7]. Most of these studies have used LAB belonging to *Leuconostoc* and *Weissella* spp. as starters for the fermentation, due to their ability to produce dextran in large amount [8].

Dextran is a homopolysaccharide with multiple applications in food products, including bakery, extrudates, and beverages [9]. LAB synthesize dextran through the action of extracellular dextransucrase (DSR) enzymes that catalyze dextran formation by cleaving the glycosidic bond of the sucrose molecule, used as a substrate, releasing glucose and fructose alongside [10]. The final dextran is a polysaccharide with at least 50% α-1,6 linked glucose as the backbone and varying percentages of α-1,4, α-1,3 and α-1,2 branched linkages [10]. The structure of dextran is mainly dependent on the type of DSR present in the bacterial strains, and on growth conditions such as sucrose amount, acidity and temperature [11]. Dextran structure and molecular weight are important features since they determine its functionality in food applications. For example, high molecular weight dextran and dextran with α-1,3 linked branches can create a polysaccharide network in the dough and improve the sensory and textural properties of bread [12, 13].

In addition to dextran, DSR can synthesize low molecular weight gluco-oligosaccharides by transferring glucose to strong acceptor molecules like maltose and isomaltose present in the substrate [10, 14]. While oligosaccharides formation hinders the efficiency of dextran formation, it can also result in a functional feature, since oligosaccharides can act as prebiotics [15, 16]. As already shown for food matrices sharing similar technological challenges, dextran biosynthesis in situ in BSG might enable its utilization in food applications such as baked goods and extrudates, conferring improved textural properties and sensory quality [7, 17, 18].

Due to the industrial importance of these enzymes, several studies in the past 20 years have investigated their functionality in different conditions. Among the approaches followed, DSR expression profile via transcription analysis has been carried out almost exclusively during bacterial growth in standard medium like MRS, or medium mimicking food conditions, and more recently during growth in wheat flour [11, 19–22]. However, understanding dextran formation mechanism in conditions relevant for industrial applications, like during food transformation, can facilitate the design of more efficient fermentation processes. The aim of this study was to assess the suitability of BSG as substrate for dextran synthesis by *Leuconostoc pseudomesenteroides* DSM20193 and *Weissella confusa* A16 previously shown as good dextran producers [23, 24] and to establish their fermentative performance. Kinetics of bacterial growth, acidification, viscosity change and metabolite formation were followed during 24 h of BSG fermentation with and without added sucrose. Differential expression of the DSR genes identified in *L. pseudomesenteroides* DSM20193 and *W. confusa* A16 during fermentation was analyzed.

## Results

### Microbial growth and acidification

Before fermentation, BSG had total presumptive LAB cell density of 1.9 ± 0.01 Log cfu/g, and the total number of aerobic mesophilic bacteria was 2.9 ± 0.4 Log cfu/g. *Bacillus cereus*, *Enterobacteriaceae*, yeasts and moulds were not detected in 10 g of BSG. After spontaneous fermentation for 24 h, cell density of presumptive LAB was 6.5 ± 1.7 Log cfu/g and aerobic mesophilic bacteria were 7.5 ± 1.4 Log cfu/g. *B. cereus* cell density was 3.9 ± 0.1 Log cfu/g. *Enterobacteriaceae* were on average 1.5 ± 2.5 Log cfu/g, and no growth of yeasts or moulds was observed. Sucrose supplementation did not affect the microbial density in spontaneously fermented BSG.

**Fig. 1:**
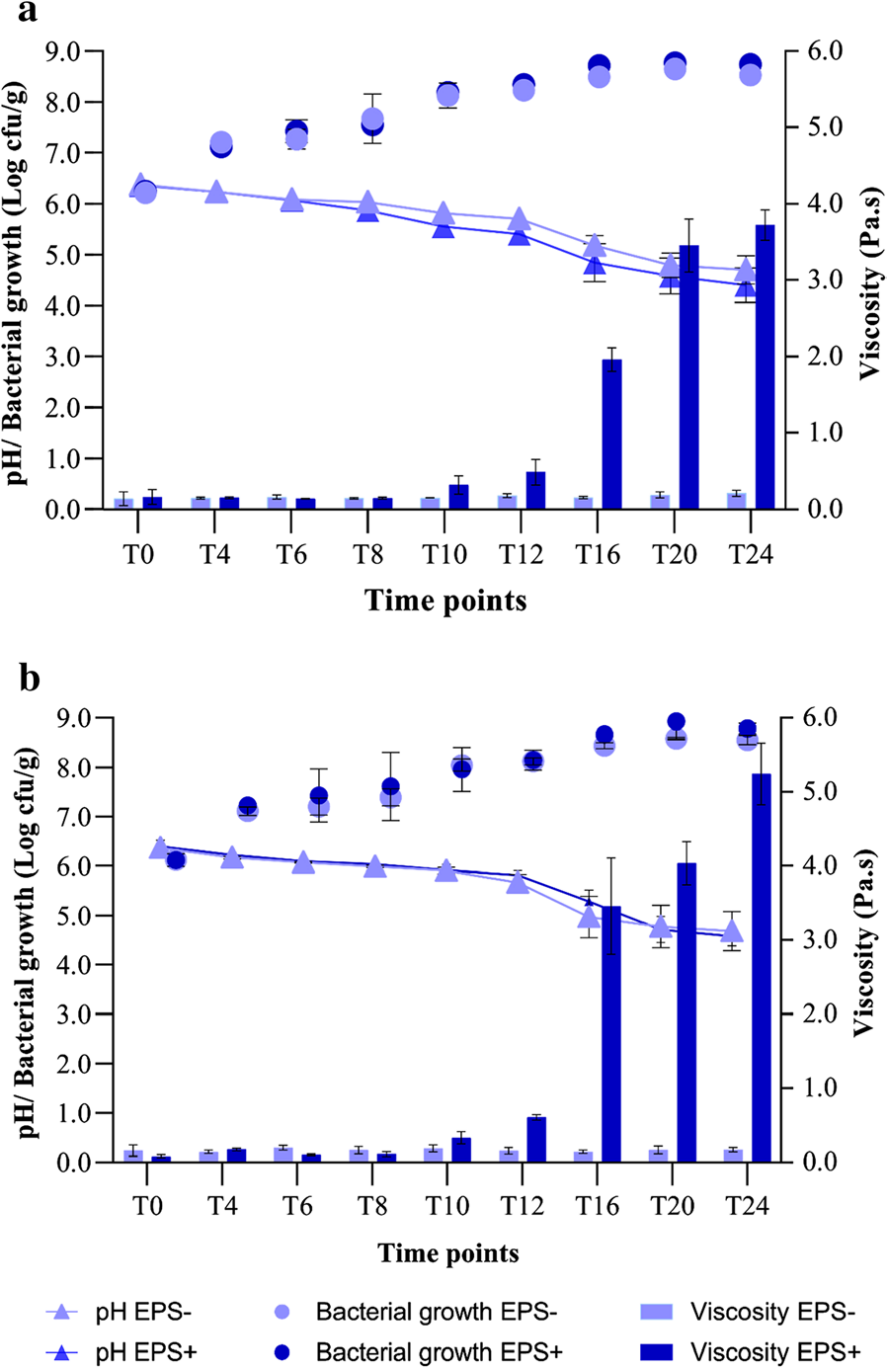
Kinetics of acidification, bacterial growth and viscosity during fermentation of EPS + and EPS− BSG. Change in pH, bacterial growth and viscosity at T0, T4, T6, T8, T10, T12, T16, T20 and T24 (hours) during fermentation of control (EPS−) and sucrose supplemented (EPS+) BSG by *Leuconostoc pseudomesenteroides* DSM20193 (**a**) and *Weissella confusa* A16 (**b**)

In controlled fermentations, the initial cell density of presumptive LAB was ca. 6.2 Log cfu/g, corresponding to the initial inoculum ratio of both the starters. Cell density in inoculated BSG increased gradually during the 24 h. At T20 cell density in BSG with or without sucrose reached ca. 8.3–8.4 Log cfu/g for both the starters and remained at similar values until 24 h (T24; Fig. 1).

The initial pH value of BSG was 6.4 ± 0.04. After 24 h of spontaneous fermentation, pH decreased to ca. 5.0 and 6.0 in BSG without and with sucrose addition, respectively. In controlled fermentation, BSG acidified consistently during the fermentation period (Fig. 1). Overall, the pH drop was slightly higher in EPS+ than EPS− for BSG fermented by *L. pseudomesenteroides* DSM20193. After 24 h, pH values were 4.7 ± 0.3 and 4.4 ± 0.3 in EPS− and EPS+ BSG fermented with *L. pseudomesenteroides* DSM20193, and 4.7 ± 0.4 and 4.6 ± 0.2 in EPS− and EPS+ BSG fermented with *W. confusa* A16.

Total titratable acidity (TTA) remained ca. 1 ml for BSG fermented spontaneously, with or without sucrose supplementation. In controlled fermentations of EPS− BSG, TTA increased in a similar manner (Table 1). After 24 h, TTA was higher for EPS + BSG fermented with *L. pseudomesenteroides* DSM20193 than with *W. confusa* A16 (3.9 and 2.7 ml, respectively). After 24 h, lactic acid content in EPS− and EPS+ BSG fermented with *L. pseudomesenteroides* DSM20193 was lower (152.7 and 131.8 mg/100 g BSG, respectively) compared to fermentation with *W. confusa* A16 (232.4 and 160.7 mg/100 g BSG, respectively). Acetic acid amount was the highest in EPS + BSG fermented with *L. pseudomesenteroides* DSM20193 throughout the fermentation time, while for BSG fermented by *W. confusa* A16 it was higher in EPS− than EPS+ BSG until T16 and reached similar values only at T24 (Table 1).

**Table 1:** TTA values and lactic and acetic acids synthesized during fermentation of EPS− and EPS + BSG

Time points	DSM20193						A16					
	TTA		Lactic acid		Acetic acid		TTA		Lactic acid		Acetic acid	
	EPS−	EPS+	EPS−	EPS+	EPS−	EPS+	EPS−	EPS+	EPS−	EPS+	EPS−	EPS+
T0	1.4 ± 0.2^a^	1.2 ± 0.1^a^	nd	nd	nd	nd	1.2 ± 0.1^a^	1.03 ± 0.1^a^	nd	nd	nd	nd
T10	1.7 ± 0.1^b^	2.2 ± 0.03^b^	45.4 ± 1.5^a^	61.7 ± 3.1^a^	3.9 ± 0.8^a^	18.1 ± 2.3^a^	1.4 ± 0.1^b^	1.5 ± 0.6^b^	47.3 ± 6.2^a^	12.1 ± 0.7^a^	7.5 ± 0.9^a^	5.9 ± 1.3^a^
T16	2.1 ± 0.01^c^	2.9 ± 0.03^c^	44.9 ± 3.7^a^	72.5 ± 4.4^b^	4.5 ± 1.2^a^	21.5 ± 1.5^a^	2.1 ± 0.1^c^	2.0 ± 0.01^c^	99.2 ± 5.8^b^	32.03 ± 2.7 ^b^	9.0 ± 1.6^a^	5.9 ± 1.1^a^
T24	2.4 ± 0.01^d^	3.9 ± 0.2^d^	152.8 ± 11.8^b^	131.8 ± 4.6^c^	4.9 ± 0.8^b^	40.2 ± 6.9^b^	2.2 ± 0.1^c^	2.7 ± 0.1^d^	225.4 ± 12.4^c^	160.7 ± 5.6 ^c^	9.4 ± 1.3^a^	9.3 ± 0.9^b^

*nd* not detected

Values of TTA (expressed as ml of 0.1 N NaOH) and amount of lactic and acetic acid (mg/100 g fermented BSG) synthesized at different time points (hours) during fermentation of control (EPS−) and sucrose supplemented (EPS+) BSG with *Leuconostoc pseudomesenteroides* DSM20193 (DSM20193) and *Weissella confusa* A16 (A16)

^a−d^Values in the same column with different letters are significantly different (Tukey’s test. P < 0.05)

### Sugars and mannitol formation

Initially (T0), 514.5 mg of glucose, 1707.7 mg of maltose, 68.7 mg of fructose, 5.9 mg of mannitol and no sucrose were found in 100 g of BSG/water mixture. During fermentation of EPS− BSG with both the starters, a similar trend of sugars consumption was observed. From T0 to T6, glucose and maltose decreased, fructose increased and remained constant until 24 h. After T10, glucose content reached 75.9 mg in EPS− BSG fermented by *L. pseudomesenteroides* DSM20193 and did not significantly change until 24 h, while some variations were seen when fermented by *W. confusa* A16 from T10 to T24. Maltose consumption was gradual during fermentation with *W. confusa* A16 unlike with *L. pseudomesenteroides* DSM20193 where it increased from T10 to T16 and decreased afterwards (Table 2)

**Table 2:** Consumption of sugars and synthesis of mannitol during fermentation of EPS− and EPS+ BSG

Starters	Time points	EPS−				EPS+				
		Glucose	Maltose	Fructose	Mannitol	Glucose	Maltose	Sucrose	Fructose	Mannitol
	T0	514.5 ± 16.4^a^	1707.7 ± 46.9^a^	68.7 ± 24.3^a^	5.9 ± 2.5^a^	514.5 ± 16.4^a^	1707.7 ± 46.9^a^	4000.0*^a^	68.7 ± 24.3^a^	5.9 ± 2.5^a^
DSM20193	T6	319.4 ± 21.1^b^	1161.6 ± 151.1^b^	95.4 ± 2.2^b^	6.1 ± 0.7^a^	173.5 ± 36.6^b^	647.4 ± 114.7^b,c^	3531.7 ± 192.1^b^	183.6 ± 9.3^b^	10.1 ± 1.1^a^
	T10	75.9 ± 3.6^c^	257.7 ± 57.1^c^	77.3 ± 3.7^c^	5.4 ± 0.9^a^	1126.1 ± 47.4^c^	980.3 ± 104.8^c^	nd	671.5 ± 83.5^c^	14.9 ± 3.6^b^
	T16	77.3 ± 8.6^c^	570.9 ± 20.2^d^	78.3 ± 3.3^c^	6.1 ± 0.9^a^	791.5 ± 9.9^a^	1731.9 ± 409.7^d^	nd	899.9 ± 152.6 ^d^	36.8 ± 4.1 ^b^
	T24	76.1 ± 7.6^c^	71.1 ± 0.7^c^	74.7 ± 0.7^c^	5.1 ± 0.5 ^a^	1499.8 ± 188.3^d^	206.5 ± 79.2^b^	nd	1085.1 ± 28.7^d^	45.2 ± 8.2^b^
A16	T6	355.4 ± 43.8^b^	1201.1 ± 259.1 ^b^	105.42 ± 5.6^b^	7.3 ± 0.4^a^	256.7 ± 73.5^b^	895.9 ± 35.6^b^	3722.9 ± 131.4^b^	288.6 ± 46.5 ^b^	8.3 ± 1.7 ^a^
	T10	84.3 ± 16.1^c^	1196.1 ± 56.9^b^	99.2 ± 8.1 ^b^	6.4 ± 0.3^ab^	148.6 ± 23.9^c^	387.4 ± 66.9^c^	nd	1357.8 ± 256.3 ^c^	6.9 ± 0.4^a^
	T16	105.5 ± 5.1^c^	1026.8 ± 41.7^b^	91.8 ± 3.3^b^	4.6 ± 1.1^c^	152.2 ± 45.5^c^	861.1 ± 39.8^b^	nd	1429.8 ± 334.6^c^	5.9 ± 0.7^b^
	T24	77.5 ± 8.7^c^	166.6 ± 27.4^c^	79.9 ± 3.6^b^	4.9 ± 0.5^bc^	119.7 ± 10.6^c^	292.9 ± 27.5^c^	nd	1652.5 ± 55.6^c^	8.4 ± 2.6^a^

*nd* not detected

Consumption of sugars and synthesis of mannitol (mg/100 g fermented BSG) at different time points (hours) in control (EPS−) and sucrose supplemented (EPS+) BSG during fermentation with *Leuconostoc pseudomesenteroides* DSM20193 (DSM20193) and *Weissella confusa* A16 (A16)

* Supplemented sucrose

^a−d^Values in the same column with different letters are significantly different (Tukey’s test. P < 0.05

.

In EPS+ BSG, supplemented sucrose was minimally utilized until 6 h and was completely consumed after 10 h of fermentation by both the starters. Fructose amount increased until T16 and remained constant afterwards (899.9 and 1429.8 mg/100 g in BSG fermented by *L. pseudomesenteroides* DSM20193 and *W. confusa* A16, respectively). After 24 h, 1.5 times more fructose was found in EPS + BSG fermented with *W. confusa* A16 than with *L. pseudomesenteroides* DSM20193. Glucose was utilized during the first 6 h by both the starters, but its consumption trend was different afterwards. Glucose increased to 1499.8 mg/100 g BSG at T24 in fermentation with *L. pseudomesenteroides* DSM20193, while it was gradually consumed by *W. confusa* A16 (reaching 119.7 mg/100 g BSG at T24). Maltose was utilized at different levels during fermentation. Maltose content was higher from T10 to T16 in EPS + BSG fermentation with *L. pseudomesenteroides* DSM20193 than with *W. confusa* A16, but it reached similar level (206.5 ± 79.2 and 292.9 ± 27.5 mg/100 g BSG) in both the fermentations after 24 h.

During fermentation of EPS− BSG, mannitol decreased to similar values 5.1 and 4.9 mg/100 g BSG in fermentation with *L. pseudomesenteroides* DSM20193 and *W. confusa* A16, respectively. In EPS + BSG fermented with *L. pseudomesenteroides* DSM20193, mannitol content increased, reaching 45.2 mg/100 g BSG at T24 while only minor changes were observed for EPS + BSG fermented with *W. confusa* A16 throughout 24 h.

### Viscosity, dextran and oligosaccharides synthesis

Viscosity values of spontaneously fermented BSG remained ca. 0.2 Pa.s for both BSG with and without sucrose supplementation, showing that no dextran was formed without the addition of the starters. Viscosity of EPS + BSG increased from 0.2 ± 0.1 (T0) to 3.7 ± 0.2 Pa.s (T24) and from 0.2 ± 0.1 (T0) to 5.5 ± 0.2 Pa.s (T24) after fermentation with *L. pseudomesenteroides* DSM20193 and *W. confusa* A16, respectively. The highest viscosity increase was observed in the interval T12-T16 (Fig. 1), corresponding to ca. 0.5 ± 0.2 to 1.9 ± 0.2 Pa.s and 0.6 ± 0.04 to 3.5 ± 0.7 Pa.s for EPS + BSG fermented with *L. pseudomesenteroides* DSM20193 and *W. confusa* A16, respectively.

In EPS– BSG, no dextran was found during fermentation with selected starters. In EPS + BSG, dextran was detected only after 10 h of controlled fermentation (Table 3). At T10, dextran amount was slightly higher in EPS + BSG fermented with *W. confusa* A16 (0.9 g/100 g) than with *L. pseudomesenteroides* DSM20193 (0.7 g/100 g fermented BSG), and it kept increasing until 24 h, reaching similar values of 1.2 and 1.1 g/100 g of BSG fermented with *L. pseudomesenteroides* DSM20193 and *W. confusa* A16, respectively.

**Table 3:** Dextran synthesis during fermentation of EPS + BSG

Time points	DSM20193	A16
T0	nd^n^	nd
T6	nd	nd
T10	0.7 ± 0.02^a,b^	0.9 ± 0.03^a^
T16	0.9 ± 0.2^b,c^	1.02 ± 0.1^c^
T24	1.2 ± 0.1^d^	1.1 ± 0.12^e^

*nd* not detected

^a−e^Values with different letters are significantly different (Tukey’s test. P < 0.05)

Amount of dextran (g/100 g BSG) synthesized during fermentation of sucrose supplemented (EPS+) BSG with *Leuconostoc pseudomesenteroides* DSM20193 (DSM20193) and *Weissella confusa* A16 (A16)

**Fig. 2:**
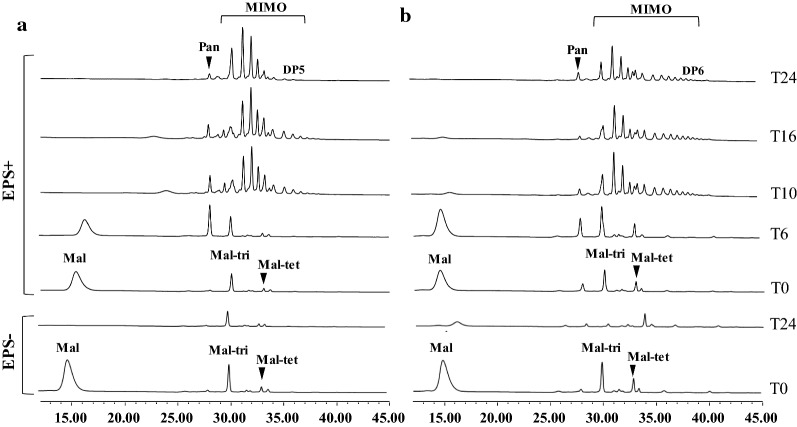
Synthesis of maltosyl-isomaltooligosaccharides (MIMO) during fermentation of EPS− and EPS+ BSG. Synthesis of MIMO by *Leuconostoc pseudomesenteroides* DSM20193 (**a**) and *Weissella confusa* A16 (**b**) during fermentation of control (EPS−) and sucrose supplemented (EPS+) BSG at T0 T6 T10 T16 and T24 (hours). Maltose (Mal). panose (Pan). maltotriose (Mal-tri) and maltotetraose (Mal-tet) are indicated in the plot. Chromatograms are plotted with HPAEC-PAD response (nC) in Y axis and retention time (min) in X axis

Regarding oligosaccharides, in EPS− BSG, maltotriose was found during fermentation with *L. pseudomesenteroides* DSM20193, while panose was detected in BSG fermented by *W. confusa* A16. In EPS + BSG, oligosaccharides were observed after T10 in both the fermentations. Overall, *W. confusa* A16 synthesized oligosaccharides with higher degree of polymerization (DP) than *L. pseudomesenteroides* DSM20193, (DP6 and DP5, respectively) for which an increase in number of peaks of oligosaccharides was observed after 24 h of fermentation (Fig. 2).

### Dextransucrases transcription analysis

To design functional quantitative PCR (qPCR) primers for genes encoding the DSRs of *L. pseudomesenteroides* DSM 20193, of which partial genomic sequence was published previously [25], the BLAST search was performed using protein sequences of glucansucrases previously identified in *Leuconostoc citreum* FDR241  strain [20]. Three candidate genes encoding proteins with reasonably high degree of homology to DSRs were identified in the genome of *L. pseudomesenteroides* DSM20193, defined as dsrD1, dsrD2 and dsrD3 with ca. 64%, 66% and 45% identity respectively to *dsrB*, *dsrA* and *dsrE* of *L. citreum* FDR241. Primers were designed to amplify two separate regions of each candidate gene and, together with primers for the reference gene (*recA* gene), used in qPCR reactions where the genomic DNA of *L. pseudomesenteroides* DSM20193 served as a template. Optimal primer pairs for each putative dextransucrase (Additional file 1: Table S1) in *L. pseudomesenteroides* DSM20193 providing ∼1:1 qPCR signal ratio towards the *recA* were identified (data not shown). For *W. confusa* A16, several candidate primer pairs designed based on the genes previously identified/annotated in genomes of other *W. confusa* strains were tested. One primers pair (Wcon-DS2-F + Wcon-DS2-R; Additional file 1: Table S1) for a DSR (defined as dsrW1) was identified in *W. confusa* A16 providing ∼1:1 qPCR signal ratio towards the *recA* (data not shown). To further validate the identity of the putative DSR in *W. confusa* A16 a partial gene sequence (accession no. MW216679) showing 92% homology with the DSR gene of *W. confusa* VTT-E90392 (Additional file 1: Fig S1) was obtained.

**Fig. 3:**
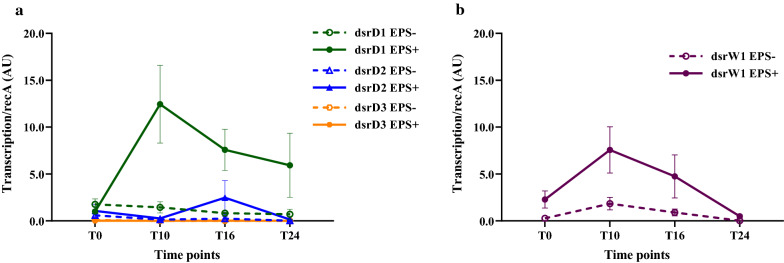
Transcriptional analysis of dextransucrase encoding genes during fermentation of EPS− and EPS+ BSG. Relative expression of three dextransucrase encoding genes (dsrD1, dsrD2 and dsrD3) present in *Leuconostoc pseudomesenteroides* DSM20193 (**a**) and one dextransucrase gene (dsrW1) present in *Weissella confusa* A16 (**b**) against housekeeping gene recA were determined at T0, T10, T16 and T24 (hours) during fermentation of control (EPS−) and sucrose supplemented (EPS+) BSG

In our study, the relative expression of DSRs was followed at selected time points during BSG fermentation, to assess the behaviour in the presence or absence of sucrose. The transcriptional analysis of *L*. *pseudomesenteroides* DSM20193 DSRs revealed differential level of expression of the three identified dsrD genes (Fig. 3). At T0, all DSRs had similar expression level in EPS− and EPS+ BSG. In all the conditions, dsrD3 remained at low expression level without any visible induction pattern. In EPS− BSG, dsrD2 followed a similar pattern, while in EPS+ BSG a small but significant upregulation was observed at T16, followed by downregulation at T24. In EPS− BSG, dsrD1 had a similar, low expression pattern. In the presence of sucrose, however, dsrD1 was significantly upregulated at T10 and then its expression slowly decreased but maintained the highest levels among the three DSRs. As above, when BSG was fermented by *W. confusa* A16, DSR expression was higher in EPS + than in EPS− BSG, and the highest expression was observed at T10. After T10, the expression gradually decreased becoming the lowest after 24 h. Overall, the expression pattern of dsrW1 was similar to dsrD1 of *L. pseudomesenteroides* DSM20193 until T16 (Fig. 3), with the difference that dsrD1 expression remained relatively high until 24 h, while dsrW1 expression significantly decreased from T16 to T24.

## Discussion

Due to its lignocellulosic composition, BSG has been mostly studied as biorefinery substrate and largely neglected by the food industry. In this study, BSG was used as a substrate for dextran biosynthesis in situ during LAB fermentation, with the aim of assessing the possibility to increase its applicability as food ingredient. The LAB starters *L. pseudomesenteroides* DSM20193 and *W. confusa* A16 were selected due to their ability to synthetize significant amount of dextran in different food substrates [13, 23]. In inoculated BSG, cell density of presumptive LAB increased of ca. 2–2.2 logarithmic cycles after 24 h, showing a clear difference compared to spontaneous fermentation, characterized by lower presumptive LAB cell density and the presence of microbial groups absent in controlled fermentations. Generally, the increasing cell density trend was similar for controlled BSG fermentations, independently of sucrose supplementation, as also observed earlier [7, 26]. For both the starters, the stationary phase could be observed after 16 h of fermentation, and the pH drop was of 1.8–1.9 units after 24 h. This indicates a relatively slow adaptation phase as compared to similar substrates (e.g. wheat and rye bran) for which higher cell density and more significant pH drop were observed after 20 h in similar fermentation conditions [6]. Generally, native BSG has limited amount of fermentable sugars but is rich in hemicellulose, cellulose, proteins and lignin [3]; thus, microbial growth will be dependent on the availability of fermentable sugars released by the spent endogenous enzymes (e.g. amylases, cellulases) and microbial enzymatic activities during fermentation. Although in beer making the saccharification process significantly reduces the free sugars content in the spent by-product, in this study, BSG had an initial amount of glucose 2–3 times higher than what observed in other cereal substrates [6, 20], while residual maltose and fructose were present, facilitating the beginning of the fermentation. Fermentation with *L. pseudomesenteroides* DSM20193 led to more acidic spent compared to *W. confusa* A16, as previously observed [13], mostly due to the capacity of *Leuconostoc* spp., typically absent in *Weissella* spp. to reduce fructose to mannitol, leading to acetic acid formation [27, 28]. In line with cell density and pH drop, also TTA and organic acids amount were slightly lower compared to similar fiber-rich matrices fermented by *W. confusa* [6, 7]. Lactic acid content was higher in EPS− than EPS + BSG and especially in fermentation with *W. confusa* A16. This could be due to different metabolism of the sugars natively available, supplemented or released during the fermentation from BSG components, potentially producing more lactic acid [29]. Dissimilar sugar consumption patterns highlighted the metabolic differences between the starters. In EPS− BSG fermentation, glucose and fructose were utilized similarly by both the strains and a slight accumulation of fructose occurred in fermentation with *L. pseudomesenteroides* DSM20193, probably due to the degradation of barley fructans [30], while maltose was utilized to a higher extent by *L. pseudomesenteroides* DSM20193. When sucrose was added to BSG, glucose, presumably deriving from fibers degradation, accumulated during fermentation with *L. pseudomesenteroides* DSM20193, while it was almost completely consumed by *W. confusa* A16. Maltose was utilized in fermentation with *L. pseudomesenteroides* DSM20193 mostly from T16 onward, and it was gradually consumed by *W. confusa* A16. Fluctuations in the level of maltose and glucose were more frequent in EPS + fermentation, probably due to DSR acceptor reactions and degradation of maltooligosaccharides. Fructose was metabolised by *L. pseudomesenteroides* DSM20193 also via the phosphoketolase pathway and was only partially utilised as electron acceptor, leading to the formation of 2.5 mmol/kg BSG after 24 h. Based on fructose liberated (2 g/100 g, corresponding to ca. 111 mmol/kg) from added sucrose, theoretically, ca. 37 to 111 mmol/kg of mannitol could have been formed, as proposed in previous studies [27, 31]. As expected, mannitol was not found in EPS + BSG fermented by *W. confusa* A16, and fructose was retrieved in concentrations close to the theoretical amount. In fermentation of lignocellulosic substrate, co-fermentation of glucose and xylose, liberated from BSG fibres, was dependent on glucose consumption rate [29, 32]. In our study, the observed sugars fluctuation at different time points, leads to hypothesize that co-fermentation of sugars happened at different stages of the fermentation, probably due to varying catabolite repression effects. This phenomenon is relatively common among LAB heterofermentative species and displays strain specific features, including a more or less relaxed repression [33, 34]. Further experiments could confirm the starters behaviour in this respect.

After 6 h of fermentation, maltose was utilised for the formation of a homologous series of maltosyl-isomaltooligosaccharides (MIMO) with α 1–6 linkages starting with panose, due to the DSR acceptor reaction [35], while maltotriose and maltotetraose might have been formed from degradation of indigenous maltooligosaccharides. The formation of IMO in cereal substrates is mostly due to the presence of acceptors such as maltose [36]. The presence and quantity of acceptor carbohydrates during cereal fermentation depend primarily on the native carbohydrate content of the cereal substrate and on its enzymatic activity [28], but also on the fermentation-induced modifications. Compared to *L. pseudomesenteroides* DSM20193, *W. confusa* A16 synthesised MIMO with higher degree of polymerization (DP5 and DP6, respectively), indicating a different activity of the microbial DSRs towards acceptor reaction [37, 38]. The structure and chain length of MIMO depend on glucansucrase specificity, on the sucrose/maltose ratio and the DSR concentration. It was previously found that, with a higher sucrose/maltose ratio, the DP of the oligosaccharides increased [39]. Thus, it is possible that in conditions of abundantly available maltose (thus lower sucrose/maltose ratio), as observed in EPS + BSG fermented by *L. pseudomesenteroides* DSM20193, the production of oligosaccharides with low DP (< 6) was favoured [37, 39]. In comparison with other cereal substrates, the DP of the MIMO in BSG remained relatively low [40]. Nonetheless, in in vitro fermentation by human fecal bacteria, maltose-based IMO with DP 5–7 showed high selectivity towards beneficial bacteria [41].

Supplemented sucrose was completely utilized during the fermentation by both the starters, and most of the synthesis occurred during the late exponential phase. In these conditions, a significant change of viscosity was observed after 10 h of fermentation in EPS + BSG. This increase happened only during controlled fermentation, clearly indicating that synthesis of dextran occurred only through the starter activity. Based on sucrose addition (4%, w/w of BSG/water mixture), 2% w/w dextran could theoretically have been formed [9]. In the conditions of this study, only ca. 55% of the theoretical dextran was synthesized, corresponding to ca. 11 g/kg of fermented BSG mass, a yield typical for cereal substrates due to DSR acceptor reactions. Besides, this amount is comparable with that previously obtained in pearl millet fermented by *W. confusa* A16 and shown to have a positive impact on bread quality when used as ingredient [24], or even higher than what found in other grains (for review see Lynch et al., 2018 [3]).

Overall, viscosity increased more gradually with *L. pseudomesenteroides* DSM20193 than with *W. confusa* A16. Although the correlation between dextran content and viscosity increase has been established in many studies, this phenomenon is not always linear [35]. The viscosity of a matrix containing dextran depends on different factors, including the different type of dextran, especially concerning structure and molar mass. As previously shown, dextran produced by *L. pseudomesenteroides* DSM 20193 and *W. confusa* A16 shares a similar structure of α 1 → 6 with α 1 → 3 branching. However, dextran from *W. confusa* A16 is more linear and of lower molar mass (up to 3% α 1 → 3 branching, 3.3 × 10^6^ g/mol g [24]) than dextran of *L. pseudomesenteroides* DSM 20193 (5.8% of α 1 → 3 branching, 4.4 × 10^6^ g/mol, [42]).

As the first increase in viscosity of the fermented BSG occurred at T10, transcriptional analysis of putative DSR genes was carried out from this time onward. One DSR gene was identified in *W. confusa* A16, and three DSR genes in *L. pseudomesenteroides* DSM20193. While *Weissella* spp. has been shown so far to possess only one DSR, *Leuconostoc* spp. can harbor several glucansucrases [43, 44]. In our conditions, dsrD1 enzyme had the highest expression among the three DSRs tested in *L. pseudomesenteroides* DSM20193 and remained active throughout the fermentation time in EPS + BSG, presumably being the main responsible for the dextran production. Similar results were observed previously during sourdough fermentation by *L. citreum* FDR241, for which only one of the five different DSRs retrieved was significantly upregulated [20]. This is also in agreement with a recent study on *L. mesenteroides* YL48 during growth on carrot medium, for which increases in dextran synthesis under high CO_2_ levels were regulated at the transcriptional level, mainly by the upregulation of one of the two DSRs found [22]. A single DSR gene identified in *W. confusa* A16 behaved similarly to dsrD1 in EPS + BSG, showing the highest expression level at T10 and slowly decreasing afterwards. Based on the time intervals considered, it appears that the major part of the enzyme was synthetized in both the starters at T10, and the synthesis was more consistent in case of *L. pseudomesenterides* DSM20193, while it showed more transient pattern in *W. confusa* A16. In previous transcriptional studies, DSR activity was detected only after several hours of contact with sucrose, showing that sucrose acts as atypical activator [19]. While DSR induction by sucrose is a common phenomenon in *Leuconostoc* spp., DSR activity of some *Weissella* spp. strains isolated from sourdough was constitutive [43]. However, in our conditions, significant upregulation of the putative DSR gene of *W. confusa* A16, previously isolated from fermented pearl millet [24], was induced only in the presence of sucrose. Although no viscosity was observed before 10 h, earlier time points could be examined in future studies to assess the gene induction pattern in more detail. On the phenotypic level, more than 50 and 85% of dextran was produced at T10, for *L. pseudomesenterides* DSM20193 and *W. confusa* A16, respectively. Overall, the dextran amount produced by the two starters was rather similar although it kept increasing at higher rates (ca. 47% from T10 to T16 and 23% from T16 to T24) in fermentation with *L. pseudomesenterides* DSM20193, than in fermentation with *W. confusa* A16 (dextran increment of 15% from T10 to T16 and 5% from T16 to T24). This difference could be due to several reasons, including different acceptor reaction and/or a different process of DSR production as indicated by the expression data. A strong upregulation of DSR gene(s) transcription was previously shown to well correlate with an increase in dextran yield during sourdough fermentations [21]. This highlights the possibility to select and use DSR genes, based on their transcription profiles, to engineer novel bacterial strains for fermentation processes, where specific DSR behavior will ensure production of desired dextran.

## Conclusions

In this study the suitability of BSG as substrate for dextran formation was assessed for the first time, showing dextran production in relevant amount from both the starters, and the synthesis of MIMO with DP > 5 potentially able to have a significant effect on BSG quality as food ingredient. Quantification of these oligosaccharides will be carried out in further studies. Furthermore, the presence of at least 3 DSR genes in *L. pseudomesenteroides* DSM20193 was shown here for the first time, one of which was most likely the main responsible for dextran synthesis. Gaining insights into the mechanism of dextran formation during BSG fermentation could contribute to a more efficient design of the fermentation process. These results illustrate a possible strategy for BSG reintegration in the food chain and studies are in progress to establish its impact in food applications.

## Materials and methods

### Microorganisms and growth conditions

*Leuconostoc pseudomesenteroides* DSM20193 was obtained from Leibniz Institute DSMZ (Braunschweig, Germany) and *W. confusa* A16 previously isolated from yellow pearl millet [24] was available at the Department of Food and Nutrition at University of Helsinki, Finland. Strains were routinely cultivated in MRS broth (Neogen, UK) at 30 °C for 24 h.

### Raw materials

Brewers’ spent grain (BSG) was provided by Viking Malt (Senson oy, Lahti, Finland) and had the following composition on dry matter basis (25.3%, AACC method 44–15.02): 19.8% protein, 2.9% ash, 9.3% fat, 55.3% dietary fiber, 9.4% carbohydrates, 0.7% glucose, 2.0% maltose, 0.1% fructose and 0.5% minerals (sodium, potassium, calcium, zinc and magnesium). Commercial granulated sugar (Suomen sokeri Oy, Finland) was used to induce the synthesis of dextran during fermentations.

### Brewers’ spent grain fermentation

For the fermentation, BSG and Milli-Q water was mixed in 2:3 ratio (referred to as EPS−). To enable dextran synthesis, 10% w/w of BSG was substituted with sucrose, resulting in 4% (w/w) sucrose on the total mixture (referred to as EPS+). LAB cells were harvested from an overnight culture by centrifugation (10,000 rpm for 10 min at room temperature) and washed once with 1X phosphate buffered saline (PBS; pH 7.4). Cell pellets were re-suspended in 1 ml of water needed for the BSG/water mixture and added to the mixture, targeting an initial cell density of 6.0 Log cfu/g. Fermentations were performed at 25 °C for 24 h. Additionally, BSG/water mixtures with and without sucrose were prepared as described above, and incubated at 25 °C for 24 h without inoculum to be used as controls.

Fermentation was performed in triplicates in batches of ca. 1000 g from which aliquots of fermented samples were withdrawn for bacterial enumeration, pH and viscosity measurement at selected time points. Kinetics of bacterial growth, acidification, metabolite formation and change in viscosity were monitored during 24 h of BSG fermentation at following time points: T0, T4, T6, T8, T10, T12, T16, T20 and T24 (in hours). Total microbial RNA was extracted from fresh aliquots of fermented BSG at 10, 16 and 24 h, and quantitative PCR analysis (qPCR) was performed.

### Bacterial enumeration, pH and total titratable acidity (TTA)

For microbial enumeration, 10 g BSG was homogenized with 90 ml of sterile 0.9% w/v NaCl solution using a stomacher (Colworth, UK), and serially diluted suspension were plated accordingly. Total aerobic mesophilic microbes, presumptive LAB, *Bacillus cereus*, *Enterobacteriaceae*, yeasts and moulds were monitored before and after 24 h of fermentation. LAB were cultivated in MRS Agar (Negoen) in micro aerophilic conditions at 30 ºC for 48 h; total aerobic mesophilic bacteria in Plate Count Agar (Neogen) 30 ºC for 72 h, and *Enterobacteriaceae* in Violet Red Bile Glucose Agar (Neogen), at 37 ºC for 24 h. *B. cereus* was cultivated in Polymyxin Pyruvate Egg-Yolk Mannitol Bromothymol Blue Agar (Neogen), at 30 ºC for 24 h, yeasts and moulds were cultivated in Yeast Extract Peptone Dextrose Agar (Neogen) and Malt Extract Agar (Neogen) both supplemented with 0.01% chloramphenicol (Oxoid, UK) at 25 ºC for 72 h and 25 ºC for 120 h respectively.

The pH of fermented BSG was measured with an online pH meter (Knick, Germany). TTA was determined using a manual titrator (Mettler Toledo DL53, Switzerland). Ten g of BSG/water mixture were mixed with 90 ml water and further homogenized using a blender (Oster, US) at full speed for 1.5 min. Titration was performed using 0.1 N NaOH up to pH 8.5. TTA values are reported as amount (ml) of 0.1 N NaOH required for titration (referred to as “ml” in the text).

### Organic acids, sugars and mannitol analysis

Amount of lactic and acetic acid was determined using high performance liquid chromatography (HPLC) system as previously explained by Xu et al. (2017). For sample preparation, 4 g of fermented and homogenized sample were mixed with Milli-Q water, vortexed for few minutes and centrifuged at 10,000 rpm for 10 min. Supernatants were syringe filtered through 0.45 µm filter (Pall, USA) for the HPLC injection.

Oligosaccharides and mannitol were analysed as explained by Xu et al. [23]. Sugars were quantified using Waters Acquity ultra performance liquid chromatography (UPLC) system fitted with evaporative light scattering detection system. For injection, 100 mg of freeze dried BSG were mixed with 2.5 ml Milli-Q water, vortexed and boiled for 10 min, centrifuged at 10,000 rpm for 10 min and supernatants were filtered through Amicon® ultra-0.5 centrifugal filter unit (Merck Millipore, Germany). Sugars were separated using Waters ACQUITY UPLC® BEH Amide 1.7 µm (2.1 × 100 mm) column, at column temperature of 35 ºC with 0.15 ml/min flow rate of mobile phases A-0.2% trimethylamine (TEA) in acetonitrile and B-0.2% TEA in Milli-Q water. The gradient applied was 85% A for 10 min, 75% A for 5 min, 55% A for 1 min and recondition for 9 min. Glucose, fructose, sucrose, maltose (Merck, Germany), panose, maltotriose, maltotetraose, maltopentaose, maltohexaose, maltoheptaose (Sigma-Aldrich, UK) and mannitol (Cerestar) were used as standards and 2-deoxy-D-galactose (Sigma-Aldrich, UK) was used as the internal standard.

### Viscosity measurement and dextran analysis

Viscosity of the fermented BSG was measured at constant shear rate of 100/s at different time points during the fermentation using rotational rheometer (Rheolab QC, Anton Paar, Germany) as explained by Xu et al. [23] with some modification. Approximately 35 g of sample were placed in C-CC27 measuring cup for 5 min and viscosity values were measured at 22 °C.

Dextran was analysed at selected time points (T0, T6, T10, T16 and T24) by an enzyme-assisted method as previously described by Katina et al. (2009) using a mixture of two enzymes, dextranase (Sigma-Aldrich, Denmark) and α-glucosidase (Megazyme, Ireland). Glucose (Merck, Germany) was used as standard and 2-deoxy-D-galactose (Sigma-Aldrich, UK) was used as the internal standard for quantification.

### Total bacterial RNA extraction, cDNA synthesis and real-time (RT) qPCR

Total bacterial RNA extraction from fermented BSG was done at selected time points T10, T16 and T24 where relative change in viscosity were the highest. As representative of T0, RNA was extracted from 24 h MRS broth culture of the strains used for inoculation. For RNA extraction from fermented BSG, 100 g of sample were collected, homogenized, and vacuum filtered with two layers of Miracloth (Millipore, MERCK). Fifty ml of filtrate was centrifuged at 500×*g* for 1 min at 4 °C. Afterwards, cell pellets were recovered from the supernatants by centrifugation at 4000×*g* for 5 min at 4 °C, re-suspended in 1 ml buffer RLT (RNeasy® Mini Kit, QIAGEN, Germany), and transferred into 2 ml screw cap tubes containing ca. 600 µl of acid-washed 425–600 µm glass beads (G8772-500G, Sigma-Aldrich). Mechanical disruption of cells was performed in FastPrep-24™ (MP Biochemicals) at max speed (6.5 m/s) for 30 s and cell lysates were centrifuged at maximum speed for 3 min at room temperature. Extraction and purification of RNA from the obtained supernatants was continued with RNeasy® Mini Kit following manufacturer’s instruction.

RNA concentration and quality were determined using a NanoDrop™ 1000 Spectrophotometer (Thermo Scientific). The cDNA was obtained by using First Strand cDNA synthesis Kit for RT-PCR (Roche) following manufacturer’s instruction. RT-PCR was performed in Stratagene® Mx3000P instrument using LightCycler 480 SYBR Green I Master Mix (Roche) according to manufacturers’ instructions. Results were analysed by the Stratagene® MxPro™ QPCR Software (Version 4.10). qPCR primer pairs for DSRs were designed with the PerlPrimer v1.1.21 software based on the DSR genes found in the genomes of *L. pseudomesenteroides* KCTC 3652 (accession numbers AEOQ00000001 to AEOQ00001160) and *W. confusa* VTT-E90392 (NZ_CP027565) available in public databases. The primers used for the analysis are listed in Additional file 1: Table S1. The thermocycling conditions were 95 °C for 30 s and 40 cycles of 95 °C for 30 s and 60 °C for 30 s. Relative gene expression profiles of DSRs under each environment at time points T0, T10, T16 and T24 was determined by comparison to the expression of the reference gene, *recA* [45]. Relative expression of DSR (DS) gene for each sample at all-time points was calculated using the cycle threshold values obtained during the qPCR with following formula, DS expression = 2^−dCT^ [46]. Partial DSR gene sequence from *W. confusa* A16 was obtained using the primer pairs reported in Additional file 1: Table S1. The partial gene sequence is deposited in GenBank under the accession number MW216679.

### Statistical analysis

Experiments were carried out as three biological replicates analysed at least two times (four times for RT qPCR). Data were subjected to two-way ANOVA and the means comparison was determined by Tukey’s test (p < 0.05) using SPSS version 25.

## Supplementary Information

**Additional file 1: Table S1.** The qPCR primers used in transcription analysis of dextransucrase genes in *L. pseudomesenteroides* DSM20193 (Lp−) and *W. confusa* A16(Wcon−) and for sequencing of dextransucrae gene of *W. confusa* A16 (Wcon-DSA16-F/R). **Figure S1.** Nucleotide of partial gene of *Weissella confusa* A16 encoding dextransucrase (accession number: MW216679) and pairwise gene alignment of partial dextransucrase encoding gene of *W. confusa* A16 (A16DS) with dextransucrase encoding gene present in *W. confusa* strain VTT E- E90392 (VTTE9).

## Data Availability

All data generated or analyses during this study are included in this published article (and its additional file).

## Abbreviations

BSG: Brewers’ spent grain
LAB: Lactic acid bacteria
DSR: Dextransucrase
MRS: De Man, Rogosa and Sharpe
TTA: Total titratable acidity
qPCR: Quantitative PCR
DP: Degree of polymerization
MIMO: Maltosyl-isomaltooligosaccharides
HPLC: High performance liquid chromatography
UPLC: Ultra performance liquid chromatography
TEA: Trimethylamine
PBS: Phosphate buffer saline
RNA: Ribonucleic acid
DNA: Deoxyribonucleic acid
cDNA: Complementary DNA
ANOVA: Analysis of variance
