# Supplementary material for: Brewers’ spent grain as substrate for dextran biosynthesis by Leuconostoc pseudomesenteroides DSM20193 and Weissella confusa A16
Source: Microb Cell Fact. 2021 Jan 22;20:23. doi: 10.1186/s12934-021-01515-4 (PMC7821685; doi:10.1186/s12934-021-01515-4)
Supplement: Supplementary file 1 — Additional file 1: Table S1. The qPCR primers used in transcription analysis of dextransucrase genes in L. pseudomesenteroides DSM20193 (Lp−) and W. confusa A16(Wcon−) and for sequencing of dextransucrae gene of W. confusa A16 (Wcon-DSA16-F/R). Figure S1. Nucleotide of partial gene of Weissella confusa A16 encoding dextransucrase (accession number: MW216679) and pairwise gene alignment of partial dextransucrase encoding gene of W. confusa A16 (A16DS) with dextransucrase encoding gene present in W. confusa strain VTT E- E90392 (VTTE9). [file 12934_2021_1515_MOESM1_ESM.docx]

**Additional file of:** Brewers’ spent grain as substrate for dextran biosynthesis by *Leuconostoc pseudomesenteroides* DSM20193 and *Weissella confusa* A16

**P. Koirala^a^, N.H. Maina^a^, H. Nihtilä^a^, K. Katina^a^, R. Coda^a,b^***

^a^ Department of Food and Nutrition, University of Helsinki, 00014 Helsinki, Finland

^b^ Helsinki Institute of Sustainability Science, Finland

**Table S1**. The qPCR primers used in transcription analysis of dextransucrase genes in *L. pseudomesenteroides* DSM20193 (Lp-) and *W. confusa* (Wcon-) and for sequencing of dextransucrae gene of *W. confusa* A16 (Wcon-DSA16-F/R)

| Primer name | sequence 5'🡪 3' | Template gene |
| --- | --- | --- |
| Lp-recA-F | CGTTTAAGGTAGCAGAAGTAGAC | recA encoding gene of *L. pseudomesenteroides* (KCTC 3652) |
| Lp-recA-R | CGTGTTCAGGATCATCAAGG |  |
| Lp-dsrD1-F | CTTTCAATCAACACCTCAGTCAG | Dextransucrase 1 encoding gene of *L. pseudomesenteroides* (KCTC 3652) |
| Lp-dsrD1-R | GCGTTTCAAATCCCAAATCGT |  |
| Lp-dsrD2-F | GGCATTACTTCTTCTCAGATGGG | Dextransucrase 2 encoding gene of *L. pseudomesenteroides* (KCTC 3652) |
| Lp-dsrD2-R | ACTGACAACCAAGTTTCCTGTG |  |
| Lp-dsrD3-F | TATCAAGTATGTTTCTGGCGG | Dextransucrase 3 encoding gene of *L. pseudomesenteroides* (KCTC 3652) |
| Lp-dsrD3-R | TGCTCAACTTCAAATCTGGG |  |
| Wcon-recA-F | GTAACCCAGAAACGACACCT | recA encoding gene of *W. confusa* |
| Wcon-recA-R | AGATTTCAACTTCCACTTCACGG |  |
| Wcon-DS2-F | CAGATTACCGTTTGATGAACCGT | Dextransucrase encoding gene of *W. confusa* VTT E-90392 |
| Wcon-DS2-R | TCGTTAGCCGTGATTGAACC |  |
| Wcon-DSA16-F | AGTACCGCCCGTTGATTGAG | Dextransucrase encoding gene of *W. confusa* A16 |
| Wcon-DSA16-R | ATGCGTGTATTCCTTAACTGCCT |  |
| Other functional qPCR primer pairs which were tested, but not selected for the transcription analysis of the dextransucrases in *L. pseudomesenteroides* and *W. confusa* | | |
| Lp-dsrD1-F2 | CTTTCAATCAACACCTCAGTCAG | Dextransucrase 1 encoding gene of *L. pseudomesenteroides* KCTC 3652 |
| Lp-dsrD1-R2 | GCGTTTCAAATCCCAAATCGT |  |
| Lp-dsrD2-F2 | GAACACAACAGTTACACCTTCAG | Dextransucrase 2 encoding gene of *L. pseudomesenteroides* KCTC 3652 |
| Lp-dsrD2-R2 | TTCCTATATTGACCAAACCATCGG |  |
| Lp-dsrD3-F2 | AAACCCAGTTGTTCAAGCAG | Dextransucrase 3 encoding gene of *L. pseudomesenteroides* KCTC 3652 |
| Lp-dsrD3-R2 | CATCACTCTTATCGACGCCA |  |
| Wcon-DS1-F | GATTGGAGTCATAACGACCCAG | Dextransucrase encoding gene of *W. cofusa* Cab3 |
| Wcon-DS1-R | CACGCACAAACGAGTAACTAGG |  |
| Other qPCR primer pairs for the dextransucrase genes found in some *W. confusa* strains, which were tested, but found less or not functional for the strain A16 | | |
| Wcon-DS3-F | AGTTGCGTGAATTGTACCCTG | *W. confusa* Cab3 |
| Wcon-DS3-R | TTCGTGTTACCCGTATTAGCC |  |
| Wcon-DS4-F | TTATCGGGATGATTATGGCTGG | *W. confusa* VTT E-90392 |
| Wcon-DS4-R | ATGACTACTATCAACTGACGCT |  |
| Wcon-DS5-F | TAACAGCTAATACAGGCAACAC | *W. confusa* VTT E-90392 |
| Wcon-DS5-R | AAGTATTGACCACCACGAACC |  |

**Figure S1**. Nucleotide of partial gene of *Weissella confusa* A16 encoding dextransucrase (accession number: MW216679) and pairwise gene alignment of partial dextransucrase encoding gene of *W. confusa* A16 (A16DS) with dextransucrase encoding gene present in *W. confusa* strain VTT E- E90392 (VTTE9)

ATGTGAATTACATGAATGATGCCTTGCAGGTATCAACTAAGTCTGTAAGTGCAGCCGATTCGGAAGCTACCCTAACCGCTGCAGCTGATGCCATTCAAGCGGCTATTGAGCGTCAGATTACGGTCAAGCAATCAACGGACTGGCTACATGAATTGATGGCAGCATTCGTTGCCACGCAACCGCAATGGAATAAGAGTAGTGAAGACGAAAATGATGATCACTTACAAGGTGGTGCGCTGTCATTTGAAAATAATTCAGATACAGATGCTAACTCCGATTACCGTTTGATGAACCGTACGCCAACTAATCAAACTGGCGACCGTTTGTACCACATTGATGATTCGTTGGGTGGCTACGAATTACTATTAGCCAATGACGTGGATAACTCAAATCCACAGGTACAAGCTGAGCAACTTAACTGGCTATACTATTTGATGAATTTTGGTTCAATCACGGCTAACGATCCGGATGCTAATTTTGATGCCATTCGTGTTGACGCTGTCGATAACGTTGATGCTGACCTATTGCAATTGGCCGCACAATATTTCCGTGAAGCGTACGGTATGGCGACAAATGATGCAACATCAAATCAGCACTTGTCAATTTTGGAAGATTGGAGTCATAACGACCCAGCCTACATGAATGACCACGGTAACGACCAATTGACGATGGATGATTACATGCATACCCAATTGATTTGGTCATTAACAAAGTCAGATGCACAACGTGGCAAGATGGATCGCTTCTTGGATTTCTACTTAACAAATCGCGCTAATGACAACACTGAAAACGAAGCTCAGCCTAGTTACTCGTTTGTGCGTGCTCACGACAGTGAAGTACAAACGGTGATTGCTGAAATTGTGACTAAGTTGCACCCTGAAGCCGGTAATGGGTTGATGCCGACCCAGGCTCAGATGGATGAAGCGTTTAAGATTTACAATGCTGATCAAAAGAAGGCAGTTAAGGAATACACGAATATTTACAACTGGAAGAGCTCAGGAAAAGAA

A16DS 1 ATGTGAATTACATGAATGATGCCTTGCAGGTATCAACTAAGTCTGTAAGTGCAGCCGATT 60

|||| ||||||||||||||||| |||||||||||||||||| ||||||||||||| || |

VTTE9 1100 ATGTAAATTACATGAATGATGCTTTGCAGGTATCAACTAAGACTGTAAGTGCAGCTGACT 1159

A16DS 61 CGGAAGCTACCCTAACCGCTGCAGCTGATGCCATTCAAGCGGCTATTGAGCGTCAGATTA 120

| |||||||| ||||| || |||||||||||||||||||| |||||||| ||||||||||

VTTE9 1160 CAGAAGCTACGCTAACTGCCGCAGCTGATGCCATTCAAGCAGCTATTGAACGTCAGATTA 1219

A16DS 121 CGGTCAAGCAATCAACGGACTGGCTACATGAATTGATGGCAGCATTCGTTGCCACGCAAC 180

|||| || ||||| ||||||||||||| |||||||||||| || ||||| | || ||||

VTTE9 1220 CGGTTAAACAATCCACGGACTGGCTACGTGAATTGATGGCGGCTTTCGTCGTGACACAAC 1279

A16DS 181 CGCAATGGAATAAGAGTAGTGAAGACGAAAATGATGATCACTTACAAGGTGGTGCGCTGT 240

||||||||||||||||||||||||| ||||| ||||||||||| |||||||||||| |||

VTTE9 1280 CGCAATGGAATAAGAGTAGTGAAGATGAAAACGATGATCACTTGCAAGGTGGTGCGTTGT 1339

A16DS 241 CATTTGAAAATAATTCAGATACAGATGCTAACTCCGATTACCGTTTGATGAACCGTACGC 300

|||||||||| ||| ||||||| |||||||| || ||||||||||||||||||||||| |

VTTE9 1340 CATTTGAAAACAATCCAGATACGGATGCTAATTCAGATTACCGTTTGATGAACCGTACAC 1399

A16DS 301 CAACTAATCAAACTGGCGACCGTTTGTACCACATTGATGATTCGTTGGGTGGCTACGAAT 360

|||| ||||||||||| || |||||||| |||||||||||||| || || || |||||||

VTTE9 1400 CAACGAATCAAACTGGTGAACGTTTGTATCACATTGATGATTCATTAGGAGGATACGAAT 1459

A16DS 361 TACTATTAGCCAATGACGTGGATAACTCAAATCCACAGGTACAAGCTGAGCAACTTAACT 420

|||||||||| ||||||||||| || |||||||| || ||||||||||||||||||||||

VTTE9 1460 TACTATTAGCGAATGACGTGGACAATTCAAATCCGCAAGTACAAGCTGAGCAACTTAACT 1519

A16DS 421 GGCTATACTATTTGATGAATTTTGGTTCAATCACGGCTAACGATCCGGATGCTAATTTTG 480

||||||| || ||||||||||||||||||||||||||||||||||| ||||| ||||| |

VTTE9 1520 GGCTATATTACTTGATGAATTTTGGTTCAATCACGGCTAACGATCCAGATGCGAATTTCG 1579

A16DS 481 ATGCCATTCGTGTTGACGCTGTCGATAACGTTGATGCTGACCTATTGCAATTGGCCGCAC 540

||||||||||||||||||| ||||||||||||||||||||||||||||||||||| || |

VTTE9 1580 ATGCCATTCGTGTTGACGCCGTCGATAACGTTGATGCTGACCTATTGCAATTGGCTGCCC 1639

A16DS 541 AATATTTCCGTGAAGCGTACGGTATGGCGACAAATGATGCAACATCAAATCAGCACTTGT 600

||||||||||||| || |||||||||||||| |||||||| ||||||||||| |||||||

VTTE9 1640 AATATTTCCGTGATGCTTACGGTATGGCGACGAATGATGCGACATCAAATCAACACTTGT 1699

A16DS 601 CAATTTTGGAAGATTGGAGTCATAACGACCCAGCCTACATGAATGACCACGGTAACGACC 660

||||||||||||||||||||||||| |||||||| ||||||||||| || || |||||||

VTTE9 1700 CAATTTTGGAAGATTGGAGTCATAATGACCCAGCTTACATGAATGAACATGGGAACGACC 1759

A16DS 661 AATTGACGATGGATGATTACATGCATACCCAATTGATTTGGTCATTAACAAAGTCAGATG 720

|||||||||||||||||||||||||||| ||||| |||||||||||||||||||||||||

VTTE9 1760 AATTGACGATGGATGATTACATGCATACACAATTAATTTGGTCATTAACAAAGTCAGATG 1819

A16DS 721 CACAACGTGGCAAGATGGATCGCTTCTTGGATTTCTACTTAACAAATCGCGCTAATGACA 780

|||| ||||| |||||||| ||||||||||||||||||||||| ||||||||||||||||

VTTE9 1820 CACAGCGTGGTAAGATGGACCGCTTCTTGGATTTCTACTTAACTAATCGCGCTAATGACA 1879

A16DS 781 ACACTGAAAACGAAGCTCAGCCTAGTTACTCGTTTGTGCGTGCTCACGACAGTGAAGTAC 840

| |||||||||||||| || ||||||||||| |||||||||||||||||||||||||| |

VTTE9 1880 ATACTGAAAACGAAGCGCAACCTAGTTACTCATTTGTGCGTGCTCACGACAGTGAAGTGC 1939

A16DS 841 AAACGGTGATTGCTGAAATTGTGACTAAGTTGCACCCTGAAGCCGGTAATGGGTTGATGC 900

|||| |||||||||||||| ||||||||||||||||||||||| || ||||| |||||||

VTTE9 1940 AAACAGTGATTGCTGAAATCGTGACTAAGTTGCACCCTGAAGCTGGGAATGGTTTGATGC 1999

A16DS 901 CGACCCAGGCTCAGATGGATGAAGCGTTTAAGATTTACAATGCTGATCAAAAGAAGGCAG 960

|||| || |||||||||||||||||||| |||||||| ||||||||||||||||||||||

VTTE9 2000 CGACTCAAGCTCAGATGGATGAAGCGTTCAAGATTTATAATGCTGATCAAAAGAAGGCAG 2059

A16DS 961 TTAAGGAATACACGAATATTTACAAC 986

|||||||||||||| | |||||||

VTTE9 2060 TTAAGGAATACACGCA---TTACAAC 2082
